# Supplementary material for: The Influence of Parental Environmental Exposure and Nutrient Restriction on the Early Life of Offspring Growth in Gambia—A Pilot Study
Source: Int J Environ Res Public Health. 2022 Oct 11;19(20):13045. doi: 10.3390/ijerph192013045 (PMC9603272; doi:10.3390/ijerph192013045)
Supplement: Supplementary file 1 [file ijerph-19-13045-s001.zip › ijerph-1811275-Supplementary.pdf]

International Journal of
Environmental Research and Public Health

MDPI

Table S1. Socio-demographic characteristics of the participants (n = 339).

Variable	Category	Frequency	Percentage
Region of Origin			
	Central River Region	14	4.1
	Upper River Region	7	2.1
	Lower River Region	16	4.7
	North Bank	42	12.4
	Banjul	9	2.7
	Western Region	182	53.7
	Kanifing Municipality Council	56	16.5
	Foreigners	13	3.8
Child alive or not			
	Alive	335	98.8
	Dead	4	1.2
Sex of child			
	Male	173	51
	Female	166	49
Marital status			
	Married	313	92.3
	Divorced	7	2.1
	Single	16	4.7
	Widow	3	0.9
Occupation of mother			
	Housewife	245	72.3
	Civil servant	38	11.2
	Farmer	6	1.8
	Self-employed	38	11.2
	Others	12	3.5
Occupation of father			
	Civil servant	212	62.5
	Farmer	28	8.3
	Construction worker	46	13.6
	Not employed	53	15.6
Residence of participant			
	Municipality regulatory area	265	78.2
	Moderate municipality regulatory area	41	12.1
	Non-municipality regulatory area	33	9.7
Settlement type			
	Urban	312	92
	Rural	27	8
Type of bathrooms			
	In house bathroom	192	56.6
	A short distance behind house	143	42.2
	Far from the house (public)	4	1.2
Highest Education completed			
	Primary	82	24.2
	Secondary	136	40.1

	Tertiary	59	17.4
	No education	62	18.3
Cooking			
	No	10	2.9
	Yes	329	97.1
Cleaning			
	No	9	2.7
	Yes	330	97.3
Laundering			
	No	17	5
	Yes	322	95

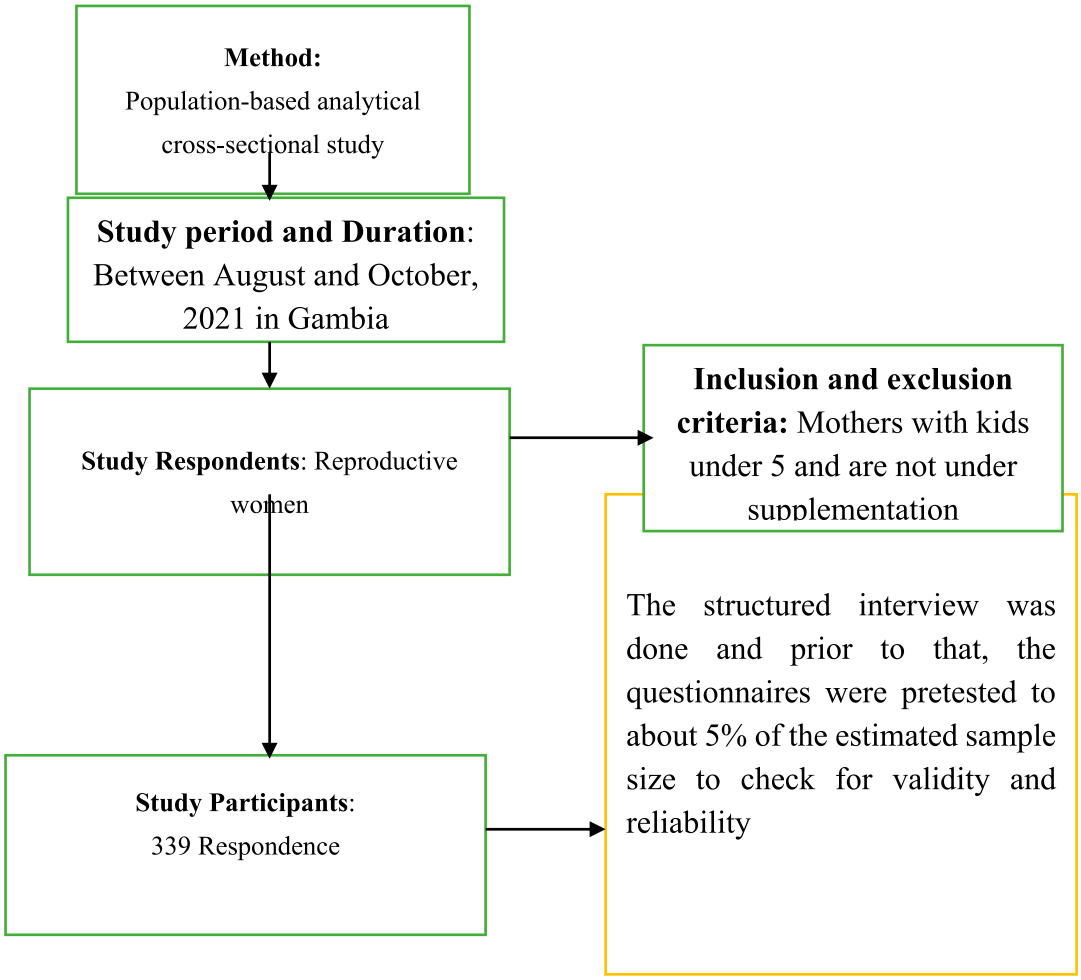

Figure S1. The Flow chart of the Experimental design.
